# Effects of mouthwashes on the morphology, structure, and mechanical properties of orthodontic materials: a systematic review of randomized clinical studies

**DOI:** 10.1093/ejo/cjaf048

**Published:** 2025-06-12

**Authors:** Angeliki Anna Gkinosati, Miltiadis A Makrygiannakis, Eleftherios G Kaklamanos

**Affiliations:** The William Harvey Research Institute - Faculty of Medicine and Dentistry, Queen Mary University of London, Charterhouse Square, London EC1M 6BQ, United Kingdom; School of Dentistry, National and Kapodistrian University of Athens, 2 Thivon str., Athens 11527, Greece; School of Dentistry, European University Cyprus, 6 Diogenous str., Engomi, Nicosia 2404, Cyprus; School of Dentistry, European University Cyprus, 6 Diogenous str., Engomi, Nicosia 2404, Cyprus; School of Dentistry, Aristotle University of Thessaloniki, Aristotle University of Thessaloniki Campus, Thessaloniki 54124, Greece; Hamdan bin Mohammed College of Dental Medicine, Mohammed bin Rashid University of Medicine and Health Sciences (MBRU), Dubai Healthcare City, P.O. Box 505055, Dubai, UAE

**Keywords:** hygiene, mechanical properties, morphology, mouthwashes, orthodontic materials, structure

## Abstract

**Background:**

Therapeutic mouthwashes are commonly used in dentistry to support mechanical plaque removal. Their interaction with orthodontic materials is increasingly studied due to potential effects on biocompatibility and biomechanics.

**Objective:**

To investigate in a systematic manner and evaluate the quality of the available evidence from randomized clinical trials regarding the effects of various mouthwashes on the morphology, structure, and mechanical properties of polymeric and metallic orthodontic materials.

**Search methods:**

Comprehensive electronic and manual searches were conducted in seven databases from inception until August 2024.

**Selection criteria:**

We analysed randomized controlled clinical studies that examined the impact of mouthwashes on the morphology, structure, and mechanical properties of both polymeric and metallic orthodontic materials.

**Data collection and analysis:**

Data eligibility, data extraction, and risk of bias (RoB-2 tool) were performed independently.

**Results:**

Ten studies were included, with most of them presenting some concerns in terms of bias. Chlorhexidine mouthrinse use did not affect the shear bond strength of polycarbonate brackets, the soldering alloys, as well as the stainless-steel bracket surfaces, but increased the roughness and friction of stainless-steel wires. The corrosion and mechanical properties of NiTi wires were not significantly affected by chlorhexidine. Rinsing with fluoride solutions increased surface roughness of NiTi wires, with more pronounced effects seen in acidulated fluoride solutions. While yield strength increased, no significant impacts on unloading force or modulus of elasticity was observed. Mouthwashes containing *Salvadora persica* did not significantly alter the surface topography of stainless-steel brackets and the roughness of stainless-steel archwires.

**Conclusions:**

The evidence from randomized clinical trials suggests that chlorhexidine- and fluoride-containing mouthwashes may impact the structure and certain mechanical properties of stainless-steel and NiTi archwires. While study limitations exist, clinicians should be mindful of possible effects on orthodontic treatment.

**Registration:**

Open Science Framework: osf.io/hz6wk

## Introduction

The risk of enamel decalcification and periodontal problems remain significant concerns in the course of orthodontic treatment, especially in individuals treated with fixed orthodontic appliances [1, 2]. Thus, a systematic and individualized oral hygiene program for every patient is of paramount significance to maintain and, if possible, improve oral health [3]. Therapeutic mouthwashes have been commonly prescribed in dentistry as an adjunct to mechanical plaque removal and, depending on the formulation, may confer benefits in terms of reduction in the prevalence and incidence of dental caries and periodontal inflammation [4].

Since mouthwashes may be administered during orthodontic treatment in individuals who have difficulty keeping plaque levels compatible with oral health through oral hygiene alone, their interaction with orthodontic materials has garnered attention, mainly due to biocompatibility and biomechanical concerns, and has been studied using various laboratory setups. The *in vitro* application of chlorhexidine-containing mouthwashes has been reported to increase both corrosion and the release of nickel ions from NiTi archwires, leading to increased surface structure porosity [5, 6]. Sodium fluoride, hydrogen peroxide and povidone iodine mouthwashes, may cause similar archwire surface changes [6–10]. Ion release and increases in surface roughness have been demonstrated to occur in brackets, both conventional and self-ligating [11–13]. The resulting increase in wire and bracket roughness could be associated with deterioration in the mechanical performance of the wire-bracket system [14–18]. Furthermore, the use of mouthwashes, especially those containing alcohol, may lead to force degradation of polymeric ligature chains [19–23]. Increased force decay has been observed following immersion in cetylpyridinium chloride-based mouthwashes [24]. Despite the body of evidence accumulated through laboratory orthodontic materials research, it should be acknowledged that laboratory designs used to mimic intraoral conditions differ significantly from the oral cavity environment [25, 26].

### Objective

The objective of the present review was to investigate in a systematic manner and evaluate the quality of the available evidence from randomized clinical trials regarding the effects of various mouthwashes on the morphology, structure, and mechanical properties of polymeric and metallic orthodontic materials.

## Materials and methods

### Protocol and registration

A specific protocol was developed and piloted following relevant guidelines [27–32]. This paper is part of a broader project which did not focus on RCTs only, whose protocol was registered in the Open Science Framework (osf.io/hz6wk). As the present study is a systematic review, ethical approval was not required.

### Eligibility criteria

The employed criteria were defined considering the Participants, Intervention, Comparison, Outcomes and Study design (PICOS) acronym domains (Supplementary Table 1). We reviewed randomized controlled clinical studies assessing the effects of mouthwashes on the morphology, structure, and mechanical properties of polymeric and metallic orthodontic materials. Animal studies, *in vitro* studies, reviews, systematic reviews, and meta-analyses were excluded.

### Information sources and search strategy

In total, seven databases (Medline (PubMed), CENTRAL (Cochrane Library; includes records from Embase, CINAHL, ClinicalTrials.gov, WHO’s ICTRP, KoreaMed, Cochrane Review Groups’ Specialized Registers), Cochrane Database of Systematic Reviews (Cochrane Library), Scopus, Web of Knowledge (including Web of Science Core Collection, KCI Korean Journal Database, Russian Science Citation Index, SciELO Citation Index, and Zoological Record), EMBASE and ProQuest Dissertation and Theses (ProQuest)) were searched up to August 2024. Also, hand-searching was employed to identify additional records. One of the authors (EGK) developed detailed search strategies for each database. They were based on the strategy developed for MEDLINE (Supplementary Table 2).

No restrictions were applied in terms of language, date, or status of publication. The reference lists in relevant articles, regardless of whether they were included or excluded, as well as other relevant articles were searched and the corresponding authors were to be contacted, if needed.

### Study selection

After exclusion of duplicates using EndNote’s duplicate identification strategy (EndNote X9™, Clarivate™, Philadelphia, PA, USA) and manually, the first two authors assessed electronically the titles and abstracts of the retrieved records for inclusion, in an independent and unblinded manner. Then, using the same approach, they obtained and evaluated the complete report of records that each reviewer considered that they were meeting the inclusion requirements. A record of all decisions on study identification was kept.

### Data collection and data items

Data extraction was performed independently by the same authors, and disagreements were again resolved through discussion or consultation with the third author. Predefined and pre-piloted data collection forms recorded the following: bibliographic details of the study, information about study design and eligibility, participant and intervention characteristics, information on study methods and outcomes, and, finally, results.

### Risk of bias in individual studies

The risk of bias in individual included studies was assessed by the same researchers in an independent manner and was evaluated using RoB2 tool for Randomized controlled trials (RCTs) [33]. Assessments were subsequently entered into the Risk-of-bias VISualization (robvis) web application [34]. Disagreements were resolved by discussion or consultation with the last author in case they were raised during any of the above-mentioned steps. Kappa statistics were not calculated since it is not recommended [32].

### Summary measures and synthesis of results, risk of bias across studies and additional analyses

Data synthesis, risk of bias across studies analyses, and additional analyses for ‘small study effects’ and publication bias were not performed due to the insufficient number of studies and the variability in study designs and outcomes [32].

## Results

### Study selection

**Figure 1.**
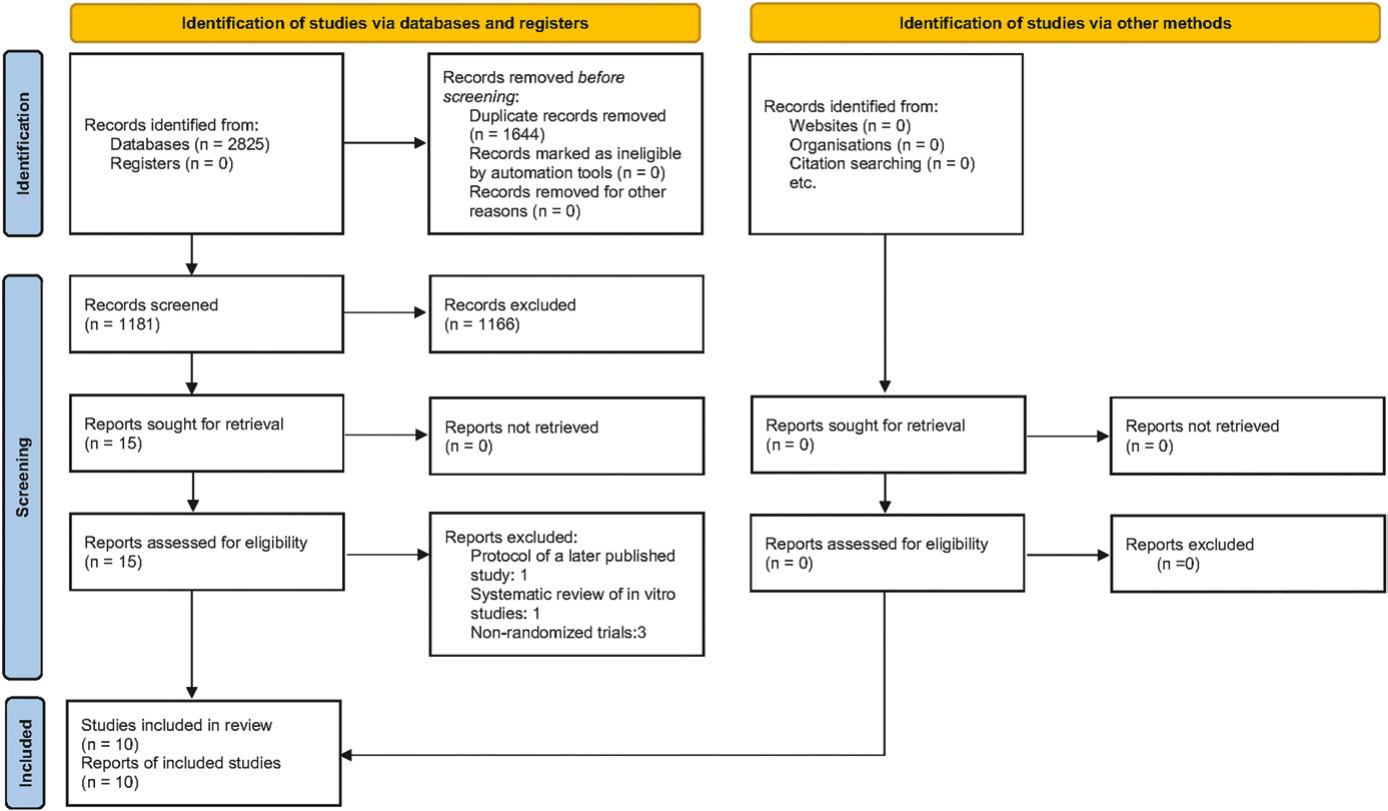
Flow of records.

We identified 2825 references, and initially excluded 1644 as duplicates and subsequently another 1166 based on their title and abstract. From the 15 records that remained and were assessed for eligibility, 5 studies were excluded for the following reasons: protocol of a later published study (n = 1), systematic review of *in vitro* studies (n = 1), non-randomized trials (n = 3) (Supplementary Table 3). Finally, 10 full-text papers were considered suitable to be included in this systematic review [35–44]. The flow of records through the reviewing process is shown in Fig. 1.

### Study characteristics

The characteristics of the included studies are shown in Table 1. The experimental period ranged from 7 days to 6 months and explored the effect of mouthwashes on polymeric adhesives [37, 41], soldering alloys [35], stainless-steel brackets and archwires [40], and NiTi archwires [36, 38, 39, 42–44], compared to control groups employing standard oral hygiene practices or using placebo. The mouthwashes tested included different concentrations of NaF (0.05 [38], 0.2% [36, 44] to 1.1% [39]), acidulated phosphate fluoride 1.1% [39], chlorhexidine (0.12% [35–37, 41–43] and 0.2% [40]), as well as a *Salvadora persica* formulation [40]. Various outcomes regarding morphology (surface topography and roughness, areas suggesting corrosion and degradation), structure (content in various elements), and mechanical properties (shear bond strength of adhesives; modulus of elasticity, hardness, yield strength, springback ratio, modulus of resilience, loading & unloading forces; friction) were reported. Measurement methodology included stereomicroscopy, energy dispersive x-ray spectroscopy, scanning electron microscopy, atomic force microscopy, cyclic polarization, electrochemical impedance spectroscopy and mechanical testing with the universal testing machine and the Nano Indenter G200.

**Table 1.** General characteristics of the studies included in the systematic review and main results.

Study—study design	Participant & intervention characteristics	Study groups	Study methods & outcomes
**Bagatin et al** [35]. 2011	**34 [14** ♂**, 20** ♀**]; Mean age:** 9y 7m [6–12y]; **Study duration:** 20d (crossbite correction) + 3m	**CG:** 17: F toothpaste [3/d] **EG:** 17; as in CG plus **0.12% CHX** [Periogard; 10mL; 1min; 2/w]	**Haas expander—soldering point areas [wire/silver brazing/band region]** [Dental Morelli] **Corrosion: [ND]** [stereomicroscopy]; Fe, Cr, O, C, P peaks **[ND]** [EDS]
**Farrag et al** [36]. 2024	**30; Age:** 18–30y; **Study duration:** 1 m	**CG:** 10; F toothpaste [3/d] **EG**_**1**_: 10; as in CG plus **0.2% NaF** [proprietary formulation; 10 mL; 2/d] **EG**_**2**_: 10; as in CG plus **0.12% CHX** [proprietary formulation; 10 mL; 2/d]	**NiTi archwires** [0.016’’×0.022’’; American Orthodontics] **Roughness:** Ra: NaF > control **[SS]**; CHX > control **[SS]** [SEM]
**Hussein et al** [37]. 2014	**18; Mean age:** 21.41 ± 1.2y [17–24y]; **Study duration:** rinsing for 7d until bonding	**PG**:9; **placebo** [20mL; 30secs; 2/d] **EG**:9; **0.12% CHX** [Peridex; 20mL; 30secs; 2/d]	**Polycarbonate brackets** [Trianeiro] bonded with **Transbond XT** [3M Unitek] **Shear bond strength:** 28d after bonding **[ND]** [UTS]
**Khanloghi et al** [38]. 2023	**10; Age:** 15–20y; **Study duration:** 6w	**CG:** 5; F^-^ toothpaste **EG:** 5; as in CG plus & **0.05% NaF** [Oral B daily; 15mL; 1/d]	**NiTi archwires - rhodium-coated** [0.016’’; GAC International] **Roughness:** Sa, Sq, Sdq increased in NAF **[SS]**; Sdr & Sz **[ND]** [AFM]
**Ogawa et al** [39]. 2020	**10 recruited [5 analysed in a cross-over trial]; Age:** 18–25y; **Study duration:** 30d for each phase [with 10d washout period between]	**PG:**5; F toothpaste [3/d] and **placebo** **EG**_**1**_: 5; as in CG but **NaF 1.1% pH 7** [proprietary formulation; 10 mL; 1/d] **EG**_**2**_: 5; as in CG but **APF 1.1% pH 5.1** [proprietary formulation; 10 mL; 1/d]	**NiTi archwires** [0.016” Nitinol, 3M Unitek & NiTi Memory Wire, American Orthodontics] **Roughness:** RA and RMS increased in APF treated Nitinol wires **[SS]**; AO wires **[ND]** [SEM] **Roughness [qualitative assessment]:** PG < NaF < APF [AFM]
**Razavi et al** [40]. 2021	**75 recruited [60 analysed]; Mean age:** 21y 5m [13–30y]; **Study duration:** 2w	**CG**: 20; F toothpaste **EG**_**1**_: 20; as in CG plus **0.20% CHX** [30mL; 30secs; 2/d] (Behsa Laboratories, Iran) **EG**_**2**_: 20; as in CG plus **Salvadora persica** [30mL; 30secs; 2/d] (Poursina Laboratories, Iran)	**S. steel brackets** [American Orthodontics] **and S. Steel archwires** (0.019” x 0.025”, 3M Unitek] **Bracket surface topography:** similar minor defects [SEM] **Archwire roughness:** Sa & Sq increased in CHX group **[SS]**; Sz, Sdr & Sbi **[ND]** [AFM] **Mechanical properties:** friction: FS **[ND]**; Fk mean **[ND]**; Fk max increased in CHX **[SS]** [UTS]
**Singh et al** [41]. 2023	**68 recruited [66 analysed; 26**♂**; 40**♀**]; Mean age:** 22.44 ± 1.34y; **Study duration:** 7d	**PG**:32; **placebo** [20mL; 30secs; 1/d] **EG**:34; **0.12% CHX** [Septodent; 20mL; 30secs; 1/d]	**Polycarbonate brackets** [manufacturer not mentioned] bonded with **Te-Econom** [Ivoclar] **Shear bond strength**: 28 days after bonding **[ND]** [UTS]
**Zibar Belasic et al** [42]. 2021	**24; Age:** 13y–42y; **Study duration:** 3 months	**CG:** 12; F toothpaste **EG: **12; 1^st^ m only—brushing without toothpaste [3/d] plus **0.12% CHX** [Curasept; 2/d]; next 2 m: as in CG	**NiTi archwires** [0.016’’×0.022’’; GAC International] **Corrosion: [ND]** [Cyclic polarization & Electrochemical impedance spectroscopy] **Roughness:** Ra, RMS & maximum height **[ND]** [AFM] **Mechanical properties:** modulus of elasticity, hardness, friction **[ND]** [Nanoindenter G200]
**Zibar Belasic et al** [43]. 2023	**67 recruited [40 analysed]; Age:** 12–22y; **Study duration:** 3m	**CG:** 20; F toothpaste **EG**:20; 1^st^ m only—brushing without toothpaste [3/d] plus **0.12% CHX** [Curasept; 2/d]; next 2 m: as in CG	**NiTi archwires **[0.020’’ x 0.020’’; GAC International] **Roughness [qualitative assessment]: [ND]** [SEM] **Mechanical properties:** modulus of elasticity, yield strength, springback ratio, modulus of resilience, loading & unloading forces **[ND]** [UTS]
**Fateh-Zonouzi et al** [44]. 2022	**20;** Age: 15–25y; **Study duration:** 6w	**CG:** 10; F toothpaste [3/d] **EG:** 10; as in CG plus **0.2% NaF** [Oral-B; 15mL; 30secs; 1/d]	**NiTi archwires - rhodium-coated** [0.016’’; GAC International] **Mechanical properties:** unloading force & modulus of elasticity [**ND**]; yield strength increased in NaF **[SS]** [UTS]

Groups receiving other interventions than mouthwashes were excluded; AFM: Atomic Force Microscopy; APF: Acidulated Phosphate Fluoride; C: Carbon; CG: control group; CHX: chlorhexidine gluconate; Cr: Chromium; d: day; EDS: Energy dispersive x-ray spectroscopy; EG: experimental group; F: fluoride; Fe: Iron; Fk: the maximum force (in N) recorded during sliding; m: months; min: minute(s); NaF: sodium fluoride; ND: No difference; NiTi: Nickel-Titanium; O: Oxygen; P: Phosphorus; PG: placebo group; Ra: Roughness Average; RMS: Root mean square; Sa: Arithmetical mean deviation of the surface; Sbi: surface bearing index; Sdr: developed surface area ratio; Sq: root-mean-square deviation; secs: seconds; SEM: Scanning Electron Microscopy; SS: statistically significant; S. Steel: stainless steel; Sz: average of ten highest and lowest points; UTS: universal testing machine; w: week; y: years; ♂: males; ♀^-^: females.

### Risk of bias within studies

**Figure 2.**
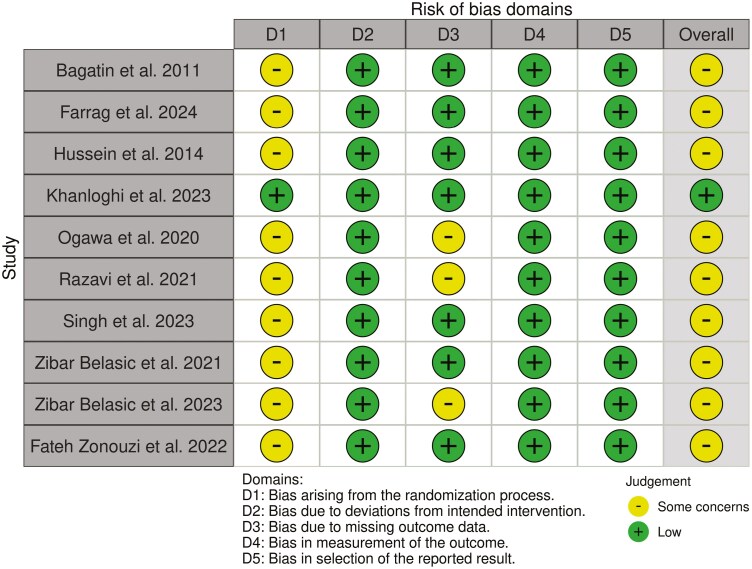
Risk of bias assessment according to ROB2 tool.

Fig. 2 summarizes the risk of bias assessment. One study was considered to have overall low risk of bias [38], while nine studies showed some concerns due to the bias arising from the randomization process [35–37, 39–44]. In addition, in 3 studies [39, 40, 42] concerns were observed regarding missing data. The cross-over study of Khanloghi et al. [38] was considered to have a low risk of bias arising from period and carryover effects.

### Results of individual studies

The use of a 0.12% chlorhexidine mouthwash for one week before bonding did not affect the shear bond strength of polycarbonate brackets bonded with composite resin adhesives compared to a placebo mouthwash [37, 41]. In comparison to brushing with fluoride toothpaste alone, additional rinses with 0.20% chlorhexidine for 2 weeks induced minor changes in the surface topography of stainless-steel brackets. However, surface roughness of stainless-steel archwires and maximum dynamic friction increased [40]. Increase in surface roughness (Ra) was demonstrated on NiTi archwires after using 0.12% chlorhexidine solutions for one month, as well [36]. However, Zibar Belasic et al. [42, 43] did not observe significant changes in their characteristics, in terms of corrosion, roughness and mechanical properties. The soldering alloys of Haas expanders in patients using a 0.12% chlorhexidine rinse for almost 4 months demonstrated similar corrosion characteristics with those brushing with fluoride toothpaste alone [35].

Daily rinses with fluoride solutions (0.05 to 1.1%) for periods ≥ 4 weeks, resulted in increases in some parameters related to surface roughness of NiTi wires, compared to brushing with fluoride toothpaste alone [36, 38] or placebo [39]. The increases were more pronounced after using acidulated fluoride solutions [39]. Yield strength of NiTi wires was reported to increase after rinsing with 0.2% NaF daily, compared to regular oral hygiene, but not statistically significant effects regarding the unloading force and the modulus of elasticity were noted [44].

Using *Salvadora persica* containing mouthwashes, did not affect stainless-steel surface bracket topography, the surface roughness of stainless-steel arch wires and the frictional characteristics compared to brushing with fluoride toothpaste alone [40] (Table 1).

## Discussion

This systematic review focuses on evaluating the quality of evidence from randomized clinical trials regarding the impact of various mouthwashes on the morphology, structure, and mechanical properties of orthodontic materials, encompassing both polymeric and metallic types. The decision to conduct such a study was made because the interaction between orthodontic materials and mouthwashes has become a subject of interest, primarily due to concerns regarding biocompatibility and biomechanical effects. Based on current evidence from randomized clinical trials, mouthwashes containing chlorhexidine and fluoride may impact the morphology and certain mechanical properties of stainless steel and NiTi archwires. The observed effects do not always corroborate the findings of laboratory investigations. Methodological dissimilarities regarding the exposure to the mouthrinse as well as the differences between experimental conditions and the oral environment may account for the discrepancies between the results derived from clinical and *in vitro* settings, as the assessment of their clinical significance [25, 26].

Chlorhexidine gluconate, a cationic bis-biguanide, possesses broad-spectrum antibacterial properties. It has long been used as a mouth rinse to combat plaque formation [42]. Chlorhexidine is effective against a broad range of microorganisms, including both gram-positive and -negative bacteria (aerobic and anaerobic) [45, 46]. Additionally, it exhibits antifungal properties, including yeasts [47]. The use of chlorhexidine mouthwashes by orthodontic patients did not influence the shear bond strength of polycarbonate brackets bonded with composite resin adhesives [37, 41], in agreement with earlier *in vitro* reports [48–50]. The chlorhexidine molecule can bind to negatively charged (anionic) surfaces, including hydroxyapatite and the acquired pellicle, but most probably this absorption phenomenon does not alter the enamel surface [50, 51]. Alternatively, acid etching may eliminate any enamel alterations affected by chlorhexidine, since application of 37% phosphoric acid for 30 s is capable of etching in a depth of 16μm [52]. Opposing observations may be attributed to differences in methodology, such as, bracket material, adhesive or etching technique used [53, 54].

Chlorhexidine mouthrinse use in the clinical setting did not affect stainless-steel bracket surfaces but increased the roughness of stainless-steel wires, as well as friction [40]. Ion release has been observed in both conventional and self-ligating brackets [12, 13], but the effects on roughness and friction from *in vitro* studies have been conflicting. Thulasiram and co-workers [11] demonstrated increases in stainless-steel bracket roughness, while Apte et al. (2025) reported no increase regarding roughness and the frictional characteristics of stainless-steel wires [55]. Corroborating were the results regarding friction observed by Himabindu et al. (2023) [56] and Hosseinzadeh Nik and co-workers [57].

The clinical effects of chlorhexidine mouthwash use on NiTi archwires have been reported to be variable. Farrag and co-workers [36] observed increases in surface roughness, while Zibar Belasic et al. [42, 43] did not observe significant changes in roughness, corrosion and the mechanical properties of NiTi archwires. *In vitro* research has shown that exposure to chlorhexidine-containing mouthwashes can induce both corrosion and nickel ion release from NiTi archwires, contributing to increased surface porosity [5, 6]. However, no statistically significant difference was observed in the average surface roughness when NiTi archwires were immersed in a chlorhexidine test environment [57]. The only study that investigated the corrosion characteristics of soldering alloys in patients using a chlorhexidine rinse did not reveal significant differences from those brushing with fluoride toothpaste alone [35]. Laboratory studies have shown the lowest amounts of ion release from silver soldering exposed to chlorhexidine solutions [58–60].

For the past five decades, fluoride mouthrinses have been widely utilized for the prevention of dental caries. The primary anti-caries benefit of fluoride is attributed to its localized action at the tooth-plaque interface. This effect is achieved by promoting the remineralization of early caries lesions and reducing the solubility of tooth enamel [61]. Routine rinsing with fluoride solutions resulted in greater surface roughness of NiTi wires [36, 38, 39], with the effect particularly noticeable with acidulated fluoride solutions [39]. Similar surface alterations of NiTi wires have been reported from *in vitro* studies [6, 8, 9, 62]. Negative impacts on various parameters associated with friction of the stainless-steel bracket- NiTi wire complex have been observed as well [15, 16, 18, 63]. Resembling observations have been made regarding the effect of fluoride mouthrinses on the release of ions from stainless-steel brackets [17] and their roughness [13], as well as the frictional characteristics of stainless-steel wires [14, 15].

Brackets and archwires are susceptible to corrosion when exposed to fluoride agents, leading to the release of metal ions into the oral environment during orthodontic treatment, with a potential of systemic absorption [64, 65]. Fluoride exposure has been shown to degrade the titanium oxide layer of NiTi archwires, contributing to pitting [29], but their corrosion is not proportional to a high fluoride content or a low pH value [66]. In clinical settings, fluoride mouthwashes have been found to primarily induce generalized corrosion rather than pitting [40, 42]. Nickel ions are released predominantly within the first three days, after which the rate of corrosion gradually declines [67]. The levels of nickel, chromium, titanium, and manganese ions in gingival crevicular fluid return to baseline approximately six months after treatment begins, though the long-term implications of metal ion release into the oral cavity require further study [64].

A general association between surface roughness and friction has been suggested, with corrosion contributing to increased roughness [68]. However, friction is also influenced by other parameters as well, such as the applied force within the wire-bracket system, the contact angle, and the bracket surface’s characteristics. A significant number of studies have investigated static and kinetic friction within the bracket-wire system using *in vitro* simulations to model orthodontic tooth movement. However, despite the extensive available research, there is still no definitive agreement on the variations in the parameters associated with friction. This lack of consensus is likely attributed to the variability and limitations in the employed research methodologies. As a result, *in vitro* studies may not serve as a dependable approach for accurately assessing the clinical impact of friction in clinical orthodontic settings [69].

With respect to the mechanical properties of NiTi wires following clinical exposure to fluoride mouthwashes, an increase in yield strength was reported. However, no statistically significant differences were observed in the unloading force or the modulus of elasticity when compared to standard oral hygiene routines [44]. Zibar Belasic and co-workers [43] showed that the load-deflection properties of NiTi wires remained unaffected after the clinical exposure to fluoride gels. Hammad and co-workers [70] observed a decrease in the modulus of elasticity of Ni-Ti wires after exposure to a fluoride solution *in vitro*, while Srivastava et al. observed no difference [71].

Corrosion does not necessarily mean that the properties of NiTi archwires will deteriorate [42]. Microstructure defects and the pH of saliva have been identified as additional possible facilitators of the corrosion of NiTi alloys, although published results vary greatly [72]. The presence of pre-existing defects could serve as initiation areas of future corrosion despite protective effects of the titanium oxide layer [73]. However, fluorides tend to dissolve the titanium oxide layer causing localized, pitting corrosion [26, 48, 74]. As a consequence, as intraoral corrosion takes place inevitably, fluoride mouthrinses may not be the decisive factors relevant to any alterations of the elastic properties of NiTi archwires. The mechanical properties of NiTi wires could possibly be influenced by the chemical interactions between fluoride formulations and the materials used in wire coatings [66]. These could account for the differing responses of rhodium-coated NiTi wires to fluoride exposure compared to uncoated wires [44, 66].

Mouthwashes containing *Salvadora persica* had no significant impact on the topography of stainless-steel brackets, the surface roughness of stainless-steel archwires, or the frictional properties when compared to brushing with fluoride toothpaste alone [40]. In fact, corrosion was higher after chlorhexidine use, suggesting *Salvadora persica*-containing mouthrinses as a better option for orthodontic patients [40]. Omidkhoda and co-workers (2017) similarly did not observe significant differences in terms of corrosion and pitting of stainless-steel and NiTi archwires exposed to a *Salvadora persica* mouthwash and artificial saliva [75]. Ion release from stainless-steel brackets immersed in a chlorhexidine mouthwash was greater compared to immersion in a *Salvadora persica* mouthwash [13].

The process of corrosion in orthodontic alloys results in the release of metal ions into the oral environment, which may have cytotoxic effects and contribute to allergic reactions in some patients. Specifically, ions such as nickel, chromium, and cobalt have been shown to impact fibroblasts and oral epithelial cells [76]. Nickel, in particular, is a common trigger for allergic reactions, which can manifest as contact dermatitis or a systemic condition [77]. Additionally, when the concentration of released ions is sufficiently high, visible enamel discolouration can occur, which can be a significant concern, as it can lead to notable aesthetic issues for patients [78].

The impact of mouthwashes on orthodontic alloy corrosion carries important biomechanical considerations that should not be overlooked. The efficiency of orthodontic treatment is closely linked to factors such as force delivery, fracture resistance, friction, and the superelasticity of NiTi alloys [79]. Superelasticity enables the consistent application of gentle forces, ensuring the resilience needed to prevent deformation within the oral environment [69]. Any alteration in the properties of NiTi archwires could lead to a reduction in fracture resistance, potentially compromising treatment efficiency. Additionally, an increase in friction levels caused by pitting or surface corrosion may hinder orthodontic tooth movement and elevate anchorage demands, further impacting the overall effectiveness of treatment [79, 80].

While the findings from clinical studies should be interpreted with caution, it is clear that the use of mouthwashes during orthodontic treatment may have significant effects. Therefore, questions regarding daily oral hygiene habits and mouthwash use should be incorporated into a patient’s dental history. If appliance corrosion is anticipated, adjustments to either the orthodontic appliances or the type of mouthwash used may be necessary to reduce the potential impact of prolonged metal ion release into the oral environment. Additionally, surface alterations could lead to increased friction, which may require modifications in anchorage preparation. To ensure the appliances remain in optimal condition, orthodontists may need to monitor patients more frequently. Furthermore, to prevent corrosion and changes in surface topography, more frequent wire replacements may be necessary.

Clinical guidelines emphasize the necessity of mouthwashes during orthodontic treatment for individuals who have difficulty keeping plaque levels compatible with oral health through oral hygiene alone, whether for a part or the entire duration of treatment [1–3]. A comprehensive risk assessment is crucial, taking into account both dental and general health factors, to improve oral hygiene, reduce plaque build-up, and avert common orthodontic issues such as gingivitis and enamel demineralization in high-risk individuals of all ages [1–3]. The recommended type of mouthwash—fluoride or antimicrobial—varies based on the patient’s needs. Nonetheless, based on the limited existing evidence, it seems that exposure to more acidic mouthwashes (e.g. acidulated phosphate fluoride versus sodium fluoride) can significantly impact the mechanical properties of orthodontic materials [39]. Therefore, it could be advisable for orthodontic patients to avoid solutions with a lower pH when low friction is necessary.

### Strengths and limitations

This systematic review was conducted in accordance with well-established guidelines, focussing solely on data from randomized controlled trials. A comprehensive search strategy was implemented, encompassing seven different databases, as well as electronic, manual, and grey literature sources up to August 2024. Strict inclusion criteria were applied to identify all eligible studies. The extracted data, study eligibility, and risk of bias assessments were independently reviewed twice by the first two authors. Any disagreements were resolved through discussion with the third author until a final consensus was reached.

Some limitations were identified in this review, primarily stemming from the characteristics of the included studies and the information available during the review process. The limited amount of relevant data, variations in study methodologies, differences in mouthwash formulations, and inconsistencies in outcome measures prevented the possibility of conducting a meta-analysis. Additionally, most studies were rated as having some concerns regarding the risk of bias, leading to uncertainty in the reliability of the findings. Also, relevant information from randomized controlled studies on popular mouthwashes, including but not limited to hydrogen peroxide, essential oils and povidone iodine mouthwashes, could not be retrieved.

Another challenge was the small sample size in several studies, further compounded by inconsistencies in mouthwash use, including variations in the control home care regimens. It is also important to consider that factors such as a patient’s overall health, oral microbiome, and prior use of mouthwash before orthodontic treatment may influence actual clinical outcomes. Moreover, the precision of the retrieved data is difficult to assess, as most studies did not include sample size calculations to determine statistical power. As a result, the extent to which different mouthwashes have a clinically significant impact on orthodontic outcomes in real-world practice remains uncertain.

### Recommendations for future research

As therapeutic mouthwashes are commonly used to support mechanical plaque removal in orthodontic patients, further clinical research is needed to investigate the potential effects on biocompatibility and biomechanics of the existing mouthrinse formulations, including but not limited to hydrogen peroxide, essential oils and povidone iodine mouthwashes, on a variety of orthodontic materials and appliances. Randomized trials of standardized designs, control of possible source of bias and research settings resembling as closely as possible to the daily clinical practice scenarios are warranted.

## Conclusion

According to the evidence from randomized clinical trials, mouthwashes containing chlorhexidine and fluoride may alter the morphology and affect certain mechanical properties of stainless-steel and NiTi archwires. Despite study limitations, clinicians should consider potential treatment-related implications.

## Supplementary Material

cjaf048_suppl_Supplementary_Tables_1-3

## Data Availability

The data underlying the results presented in the study are available in the original publications that were included in this study.

## References

[1] Graber TM , Eliades T, Athanasiou AE. Risk Management in Orthodontics: Experts’ Guide to Malpractice. Chicago: Quintessence Publishing Co., 2004.

[2] Al Makhmari SA , Kaklamanos EG, Athanasiou AE. Short-term and long-term effectiveness of powered toothbrushes in promoting periodontal health during orthodontic treatment: A systematic review and meta-analysis. Am J Orthod Dentofacial Orthop 2017;152:753–66.e7. https://doi.org/10.1016/j.ajodo.2017.09.003 29173855

[3] Heintze SD , Jost-Brinkmann PG, Finke C, Miethke RR. Oral health for the orthodontic patient. Berlin, Germany: Quintessence, 1999.

[4] Mariotti AJ , Burrell KH. Mouthrinses and dentifrices (5th ed.). Chicago: American Dental Association and Physician’s Desk Reference, Inc. 2009.

[5] Ramya P , Raghu Ram R, Ranganayakulu I, et al Effect of charcoal, probiotic, and chlorhexidine mouthwashes on mechanical properties and surface characterization of ceramic-coated nickel-titanium orthodontic arch wires: a comparative in-vitro study. Cureus 2023;15:e40791. https://doi.org/10.7759/cureus.40791 37485112 PMC10362787

[6] Sufarnap E , Harahap KI, Adiana ID, et al Corrosion of copper nickel titanium archwire in chlorhexidine, sodium fluoride, and chitosan mouthwashes. F1000Research 2024;12:159. 38434650 10.12688/f1000research.129043.3 PMC10905017

[7] Jothy K. Evaluation of the effects of povidone iodine and hydrogen peroxide mouthwashes on orthodontic Archwires: an *In vitro* Study. J Contemp Dent Pract 2023;24:228–37. https://doi.org/10.5005/jp-journals-10024-3484 37469261

[8] Sukumar B , Raghu Ram R, Sunil G, et al Effect of sodium Flouride mouthwash and ozone-infused oil pulling solution with coconut oil on mechanical properties and surface characterization of copper-nickel-titanium orthodontic Archwires. Cureus 2023;15:e40207. https://doi.org/10.7759/cureus.40207 37435266 PMC10332333

[9] Pastor F , Rodriguez JC, Barrera JM, et al Effect of fluoride content of mouthwashes on the metallic ion release in different orthodontics archwires. Int J Environ Res Public Health 2023;20:2780. https://doi.org/10.3390/ijerph20042780 36833476 PMC9956897

[10] Mirhashemi A , Jahangiri S, Kharrazifard M. Release of nickel and chromium ions from orthodontic wires following the use of teeth whitening mouthwashes. Prog Orthod 2018;19:4. https://doi.org/10.1186/s40510-018-0203-7 29399703 PMC5797726

[11] Thulasiram SRSR , Chandrasekharan D, Angrish N, et al Comparison of surface topography changes from conventional metal brackets and self-ligating metal brackets after immersion in three different mouthwashes using atomic force microscopy: an *in vitro* study. J Pharm Bioallied Sci 2023;15:S636–40. https://doi.org/10.4103/jpbs.jpbs_78_23 37654337 PMC10466524

[12] Nahidh M , Garma NM, Jasim ES. Assessment of ions released from three types of orthodontic brackets immersed in different mouthwashes: an *in vitro* study. J Contemp Dent Pract 2018;19:73–80. https://doi.org/10.5005/jp-journals-10024-2214 29358538

[13] Danaei SM , Safavi A, Roeinpeikar SM, et al Ion release from orthodontic brackets in 3 mouthwashes: An in-vitro study. Am J Orthod Dentofacial Orthop 2011;139:730–4. https://doi.org/10.1016/j.ajodo.2011.03.004 21640878

[14] Nik TH , Ghadirian H, Hooshmand T, et al Effect of 0.05% sodium fluoride mouthwash on surface roughness and friction between ceramic brackets and rhodium-coated and uncoated stainless steel wires. Front Dent 2019;16:121–9. https://doi.org/10.18502/fid.v16i2.1363 31777853 PMC6874846

[15] Geramy A , Hooshmand T, Etezadi T. Effect of sodium fluoride mouthwash on the frictional resistance of orthodontic wires. J dent (Tehran) 2017;14:254–8. 29296110 PMC5748452

[16] Schiff N , Boinet M, Morgon L, et al Galvanic corrosion between orthodontic wires and brackets in fluoride mouthwashes. Eur J Orthod 2006;28:298–304. https://doi.org/10.1093/ejo/cji102 16428255

[17] Schiff N , Dalard F, Lissac M, et al Corrosion resistance of three orthodontic brackets: a comparative study of three fluoride mouthwashes. Eur J Orthod 2005;27:541–9. https://doi.org/10.1093/ejo/cji050 16049037

[18] Schiff N , Grosgogeat B, Lissac M, et al Influence of fluoridated mouthwashes on corrosion resistance of orthodontics wires. Biomaterials 2004;25:4535–42. https://doi.org/10.1016/j.biomaterials.2003.11.042 15120498

[19] Castelló CA , Zamora-Martínez N, Paredes-Gallardo V, et al Effect of mouthwashes on the force decay of polymeric ligature chains used for dental purposes: a systematic review and meta-analysis. BMC Oral Health 2023;23:538. https://doi.org/10.1186/s12903-023-03240-3 37542215 PMC10401800

[20] Javidi P , Bashardoust N, Shekarbaghani A. Evaluation of force decay rate in orthodontic elastomeric chains in the environment of various mouthwashes: a systematic review. J Dent Res 2023;20:39. PMC10166759 37180681

[21] Issa AR , Kadhum AS, Mohammed SA. The effects of zinc-containing mouthwashes on the force degradation of orthodontic elastomeric chains: an *In vitro* study. Int J Dent 2022;2022:3557317. https://doi.org/10.1155/2022/3557317 35531573 PMC9076336

[22] Behnaz M , Namvar F, Sohrabi S, et al Effect of bleaching mouthwash on force decay of orthodontic elastomeric chains. J Contemp Dent Pract 2018;19:221–5. https://doi.org/10.5005/jp-journals-10024-2240 29422474

[23] Pithon MM , Rodrigues AC, Sousa EL, et al Do mouthwashes with and without bleaching agents degrade the force of elastomeric chains? Angle Orthod. 2013;83:712–7. 23311601 10.2319/081012-646.1 PMC8754043

[24] Castelló C , Zamora-Martínez N, Tarazona-Álvarez B, et al Comparative evaluation of two cetylpyridinium chloride-based mouthwashes on the mechanical properties and strength loss of elastomeric chains used in dentistry: An vitro study. Heliyon 2024;10:e27721. 38545197 10.1016/j.heliyon.2024.e27721 PMC10965520

[25] Eliades T , Bourauel C. Intraoral aging of orthodontic materials: the picture we miss and its clinical relevance. Am J Orthod Dentofacial Orthop 2005;127:403–12. https://doi.org/10.1016/j.ajodo.2004.09.015 15821684

[26] Eliades T , Athanasiou AE. In vivo aging of orthodontic alloys: implications for corrosion potential, nickel release, and biocompatibility. Angle Orthod 2002;72:222–37. https://doi.org/10.1043/0003-3219(2002)072<0222:IVAOOA>2.0.CO;2 12071606

[27] Moher D , Shamseer L, Clarke M, et al; PRISMA-P Group. Preferred reporting items for systematic review and meta-analysis protocols (PRISMA-P) 2015 statement. Syst Rev 2015;4:1. https://doi.org/10.1186/2046-4053-4-1 25554246 PMC4320440

[28] Shamseer L , Moher D, Clarke M, et al; the PRISMA-P Group. Preferred reporting items for systematic review and meta-analysis protocols (PRISMA-P) 2015: elaboration and explanation. BMJ 2015;349:g7647–g7647. https://doi.org/10.1136/bmj.g7647 25555855

[29] Page MJ , McKenzie JE, Bossuyt PM, et al The PRISMA 2020 statement: an updated guideline for reporting systematic reviews. BMJ 2021;372:n71. https://doi.org/10.1136/bmj.n71 33782057 PMC8005924

[30] Rethlefsen ML , Kirtley S, Waffenschmidt S, et al; PRISMA-S Group. PRISMA-S: an extension to the PRISMA Statement for Reporting Literature Searches in Systematic Reviews. Syst Rev 2021;10:39. https://doi.org/10.1186/s13643-020-01542-z 33499930 PMC7839230

[31] Rethlefsen ML , Page MJ. PRISMA 2020 and PRISMA-S: common questions on tracking records and the flow diagram. JMLA 2022;110:253–7. https://doi.org/10.5195/jmla.2022.1449 35440907 PMC9014944

[32] Higgins JPT , Thomas J, Chandler J, Cumpston M, Li T, Page MJ, Welch VA. (Eds.). Cochrane Handbook for Systematic Reviews of Interventions version 6.5. Cochrane. 2024. www.training.cochrane.org/handbook

[33] Sterne JAC , Savović J, Page MJ, et al RoB 2: a revised tool for assessing risk of bias in randomised trials. BMJ 2019;366:l4898. https://doi.org/10.1136/bmj.l4898 31462531

[34] McGuinness LA , Higgins J. Risk-of-bias VISualization (robvis): An R package and Shiny web app for visualizing risk-of-bias assessments. Res Synth Methods 2021;12:55–61. 32336025 10.1002/jrsm.1411

[35] Bagatin CR , Ito IY, Andrucioli MC, et al Corrosion in Haas expanders with and without use of an antimicrobial agent: an in situ study. J Appl Oral Sci 2011;19:662–7. https://doi.org/10.1590/s1678-77572011000600020 22231004 PMC3973471

[36] Farrag OGAEG , Shamaa NEA, Elgameay WE, et al Clinical effect of chlorhexidine and sodium fluoride on corrosion behavior and surface topography of nitinol orthodontic archwires. BMC Oral Health 2024;24:564. https://doi.org/10.1186/s12903-024-04289-4 38745154 PMC11092164

[37] Hussein FA , Hashem MI, Chalisserry EP, et al The impact of chlorhexidine mouth rinse on the bond strength of polycarbonate orthodontic brackets. J Contemp Dent Pract 2014;15:688–92. https://doi.org/10.5005/jp-journals-10024-1600 25825091

[38] Khanloghi M , Sheikhzadeh S, Khafri S, et al Effect of different forms of fluoride application on surface roughness of rhodium-coated NiTi orthodontic wires: a clinical trial. Front dent 2023;20:13. https://doi.org/10.18502/fid.v20i13.12660 37312830 PMC10258391

[39] Ogawa CM , Faltin K Jr., Maeda FA, et al In vivo assessment of the corrosion of nickel-titanium orthodontic archwires by using scanning electron microscopy and atomic force microscopy. Microsc Res Tech 2020;83:928–36. https://doi.org/10.1002/jemt.23486 32233101

[40] Razavi EE , Nik TH, Hooshmand T, et al Surface characterization and frictional force between stainless steel brackets and archwires in orthodontic patients using chlorhexidine- and Persica-containing mouthrinses: A randomized controlled trial. J Dent Res 2021;18:21. PMC8248265 34249247

[41] Singh J , Kumar A, Gupta E, et al Evaluation of the impact of chlorhexidine mouth rinse on the bond strength of polycarbonate orthodontic brackets: a case-control study. Cureus 2023;15:e38227. https://doi.org/10.7759/cureus.38227 37261189 PMC10226845

[42] Zibar Belasic T , Pejova B, Curkovic HO, et al Influence of intraoral application of antiseptics and fluorides during orthodontic treatment on corrosion and mechanical characteristics of nickel-titanium alloy in orthodontic appliances. Angle Orthodontics 2021;91:528–37. 10.2319/052620-480.1 PMC8259758 33566077

[43] Zibar Belasic T , Zigante M, Uhac M, et al Effect of use of antiseptics and fluorides during orthodontic treatment on working properties of NiTi archwires in levelling dental arches: A randomized controlled trial. J Orofac Orthop 2023;85:63–72. https://doi.org/10.1007/s00056-023-00475-1 37358625

[44] Zonouzi FM , Kamel RM, Sheikhzadeh S, et al The effect of different methods of fluoride administration at different concentrations on the load-deflection properties of rhodium-coated niti archwires. J Babol Univ Med Sci 2022;24:25–32.

[45] Emilson CG. Susceptibility of various microorganisms to chlorhexidine. Scand J Dent Res 1977;85:255–65. https://doi.org/10.1111/j.1600-0722.1977.tb00561.x 266752

[46] Matthijs S , Adriaens PA. Chlorhexidine varnishes: A review. J Clin Periodontol 2002;29:1–8. https://doi.org/10.1034/j.1600-051x.2002.290101.x 11846842

[47] Puig Silla M , Montiel Company JM, Almerich Silla JM. Use of chlorhexidine varnishes in preventing and treating periodontal disease: A review of the literature. Med Oral Patol Oral Cir Bucal 2008;13:E257–60. 18379452

[48] Damon PL , Bishara SE, Olsen ME, et al Bond strength following the application of chlorhexidine on etched enamel. Angle Orthod 1997;67:169–72. https://doi.org/10.1043/0003-3219(1997)067<0169:BSFTAO>2.3.CO;2 9188959

[49] Rajendran A , Sundareswaran S, Peediyekkal LV, et al Effect of oral environment and prescribed fluoride mouthwashes on different types of TMA wires - An in-vivo study. J Orthod Sci 2019;8:8. https://doi.org/10.4103/jos.JOS_72_18 31161131 PMC6540775

[50] Demir A , Malkoc S, Sengun A, et al Effects of chlorhexidine and povidone-iodine mouthrinses on the bond strength of an orthodontic composite. Angle Orthod. 2005;75:392–6. 15898378 10.1043/0003-3219(2005)75[392:EOCAPM]2.0.CO;2

[51] Jenkins S , Addy M, Wade W. The mechanism of action of chlorhexidine: A study of plaque growth on enamel inserts in vivo. J Clin Periodontol 1988;15:415–24. https://doi.org/10.1111/j.1600-051x.1988.tb01595.x 3183067

[52] Legler LR , Retief DH, Bradley EL. Effects of phosphoric acid concentration and etch duration on enamel depth of etch: an *in vitro* study. Am J Orthod Dentofacial Orthop 1990;98:154–60. https://doi.org/10.1016/0889-5406(90)70009-2 2198801

[53] Catalbas B , Ercan E, Erdemir A, et al Effects of different chlorhexidine formulations on shear bond strengths of orthodontic brackets. Angle Orthod 2009;79:312–6. https://doi.org/10.2319/032008-158.1 19216606

[54] Polat O , Uysal T, Karaman AI. Effects of a chlorhexidine varnish on shear bond strength in indirect bonding. Angle Orthod 2005;75:1036–40. https://doi.org/10.1043/0003-3219(2005)75[1036:EOACVO]2.0.CO;2 16448252

[55] Apte S , Divya S, Urala AS. Impact of chlorine dioxide and chlorhexidine mouthwashes on friction and surface roughness of orthodontic stainless steel wires: An in-vitro comparative study. F1000Research 2025;13:1442. 39850614 10.12688/f1000research.158974.2 PMC11754953

[56] Himabindu D , Venkata Prasanna P, Vamsi Krishna Reddy V, et al Influence of different mouth rinsing agents on friction during sliding mechanics between orthodontic metal brackets and stainless steel archwire: A comparative in vitro study. Cureus 2023;15:e41224. https://doi.org/10.7759/cureus.41224 37525764 PMC10387376

[57] Hosseinzadeh NT , Hooshmand T, Farazdaghi H, et al Effect of chlorhexidine-containing prophylactic agent on the surface characterization and frictional resistance between orthodontic brackets and archwires: An *in vitro* study. Prog Orthod 2013;14:48. 24325758 10.1186/2196-1042-14-48 PMC3895700

[58] Saturno Corrêa da Costa C , Neves JG, Borges LPS, et al Comparison of the physico-chemical impact of chlorhexidine and silver nanoparticles on orthodontic appliances made with laser and silver solder: An in vitro study. Int. Orthod 2022;20:100631. https://doi.org/10.1016/j.ortho.2022.100631 35272970

[59] Shetti SS , Shirkhande A, Kagi VA, et al The effect of different mouthwashes on metallic ions release from silver-soldered and laser-welded orthodontic attachments: A comparative in vitro study. Dent. Res. J. 2022;19:27. PMC9006156 35432787

[60] Erdogan AT , Nalbantgil D, Ulkur F, et al Metal ion release from silver soldering and laser welding caused by different types of mouthwash. Angle Orthod 2015;85:665–72. https://doi.org/10.2319/050914-335.1 25191838 PMC8611754

[61] Featherstone JDB , Ten Cate JM. Physicochemical aspects of fluoride-enamel interactions. In Ekstrand J, Fejerskov O, Silverstone LM. (Eds.), Fluoride in dentistry. Copenhagen: Munksgaard, 1988, pp. 125–49.

[62] Huang HH. Variation in surface topography of different NiTi orthodontic archwires in various commercial fluoride-containing environments. Dent. Mater 2007;23:24–33. https://doi.org/10.1016/j.dental.2005.11.042 16417915

[63] Kao CT , Ding SJ, Wang CK, et al Comparison of frictional resistance after immersion of metal brackets and orthodontic wires in a fluoride-containing prophylactic agent. Am J Orthod Dentofacial Orthop 2006;130:568.e1–9. https://doi.org/10.1016/j.ajodo.2005.09.028 17110251

[64] Chitra P , Prashantha GS, Rao A. Long-term evaluation of metal ion release in orthodontic patients using fluoridated oral hygiene agents: an in vivo study. J World Fed Orthod 2019;8:107–11. https://doi.org/10.1016/j.ejwf.2019.04.003

[65] Chitra P , Prashantha GS, Rao A. Effect of fluoride agents on surface characteristics of NiTi wires: an ex vivo investigation. J Oral Biol Craniofac Res 2020;10:435–40. https://doi.org/10.1016/j.jobcr.2020.07.006 32817814 PMC7426570

[66] Katić V , Mandić V, Ježek D, et al Influence of various fluoride agents on working properties and surface characteristics of uncoated, rhodium coated and nitrified nickel-titanium orthodontic wires. Acta Odontol Scand 2015;73:241–9. 25643671 10.3109/00016357.2014.980847

[67] Rincic Mlinaric M , Karlovic S, Ciganj Z, et al Oral antiseptics and nickel-titanium alloys: mechanical and chemical effects of interaction. Odontology 2019;107:150–7. https://doi.org/10.1007/s10266-018-0387-9 30178177

[68] Costa MT , Lenza MA, Gosch CS, et al *In vitro* evaluation of corrosion and cytotoxicity of orthodontic brackets. J Dent Res 2007;86:441–5. https://doi.org/10.1177/154405910708600510 17452565

[69] Eliades T , Brantley, W. (Eds.). Orthodontic Applications of Biomaterials: A Clinical Guide. Amsterdam: Elsevier, 2017, 97–104.

[70] Hammad SM , Al-Wakeel EE, Gad E-S. Mechanical properties and surface characterization of translucent composite wire following topical fluoride treatment. Angle Orthodontics 2012;82:8–13. 10.2319/030811-168.1 PMC8881028 21721949

[71] Srivastava K , Chandra PK, Kamat N. Effect of fluoride mouth rinses on various orthodontic archwire alloys tested by modified bending test: an *in vitro* study. Indian J Dent Res 2012;23:433–4. https://doi.org/10.4103/0970-9290.102253 23059594

[72] Močnik P , Kosec T. A Critical appraisal of the use and properties of nickel-titanium dental alloys. Materials (Basel) 2021;14:7859. https://doi.org/10.3390/ma14247859 34947453 PMC8703947

[73] Cacciafesta V , Sfondrini MF, Stifanelli P, et al Effect of chlorhexidine application on shear bond strength of brackets bonded with a resin-modified glass ionomer. Am J Orthod Dentofacial Orthop 2006;129:273–6. https://doi.org/10.1016/j.ajodo.2004.07.050 16473721

[74] Walker MP , Ries D, Kula K, et al Mechanical properties and surface characterization of beta titanium and stainless steel orthodontic wire following topical fluoride treatment. Angle Orthod 2007;77:342–8. https://doi.org/10.2319/0003-3219(2007)077[0342:MPASCO]2.0.CO;2 17319772

[75] Omidkhoda M , Poosti M, Sahebnasagh Z, et al Effects of three different mouthwashes on the surface characteristics of nickel-titanium and stainless steel archwires in orthodontics. J Dent Mater Tech 2017;6:19–266.

[76] Schmalz G , Garhammer P. Biological interactions of dental cast alloys with oral tissues. Dent. Mater 2002;18:396–406. https://doi.org/10.1016/s0109-5641(01)00063-x 12175579

[77] Bodén K , Schenker M, Lidén C. Oral cytotoxicity of orthodontic metallic materials: An experimental study. Eur J Orthod 2016;38:400–6.

[78] Singh N , Sharma S, Saimbi CS. Allergic reactions to nickel in orthodontics: Implications and management. J Clin Orthod 2021;55:153–8.

[79] Proffit, W. R., Fields, H. W., Larson, B. E., & Sarver, D. M. Contemporary orthodontics (6th ed.). Amsterdam: Elsevier, 2019.

[80] Heravi F , Mokhber N, Shayan E. Galvanic corrosion among different combinations of orthodontic archwires and stainless steel brackets. J Dent Mater Tech 2014;3:118–22.

