# Supplementary material for: Effects of mouthwashes on the morphology, structure, and mechanical properties of orthodontic materials: a systematic review of randomized clinical studies
Source: Eur J Orthod. 2025 Jun 12;47(4):cjaf048. doi: 10.1093/ejo/cjaf048 (PMC12159413; doi:10.1093/ejo/cjaf048)
Supplement: cjaf048_suppl_Supplementary_Tables_1-3 [file cjaf048_suppl_supplementary_tables_1-3.docx]

**Supplementary Table 1.** Eligibility criteria.

| **Domain** | **Inclusion criteria** | **Exclusion criteria** |
| --- | --- | --- |
| **Participants** | • Human subjects undergoing any kind of orthodontic treatment (with the retrieval of orthodontic appliances and / or accessories performed during or at the end of treatment). |  |
| **Interventions** | • Use of any mouthwash at any concentration. | • Concurrent administration of substances in formulations other than mouthwashes, toothpastes, gels, etc. (in the context of usual oral hygiene) |
| **Comparisons** | • Placebo intervention (preferably) or no intervention/usual oral hygiene. | • Comparison to other mouthwashes, without a placebo or control group |
| **Outcomes** | • Qualitative and quantitative data regarding the effects of different mouthwashes on effects of various mouthwashes on the morphology, structure, and mechanical properties of polymeric and metallic orthodontic materials [Stereomicroscopy, Scanning electron microscopy, Energy dispersive X-ray spectroscopy, three bending tests etc.]. |  |
| **Study design** | • Randomized controlled studies (according to the Scottish Intercollegiate Guidelines Network algorithm for classifying study design (https://www.sign.ac.uk/media/1610/sign50study_design.pdf). | • *In vitro* studies. Animal studies. Reviews, systematic reviews and meta-analyses.  • Less than 5 subjects per group analysed (Mead, 2012). |

Mead, R., Gilmour S. G., & Mead, A. (2012). Statistical Principles for the Design of Experiments. Cambridge: Cambridge University Press.

**Supplementary Table 2.** Strategy for database search.

| **Database [2024 08 16]** | **Search strategy** | **Hits** |
| --- | --- | --- |
| **PubMed** | (chlor*[tiab] OR mouthrinse[tiab] OR mouthwash[tiab] OR mouth-rinse[tiab] OR "mouth rinse"[tiab] OR mouth-wash[tiab] OR "mouth wash"[tiab]) AND orthodont*[tiab] | **496** |
| **Cochrane Central Register of Controlled Trials** | (chlor* OR mouthrinse OR mouthwash OR mouth-rinse OR "mouth rinse" OR mouth-wash OR "mouth wash") AND orthodont* in Record Title OR (chlor* OR mouthrinse OR mouthwash OR mouth-rinse OR "mouth rinse" OR mouth-wash OR "mouth wash") AND orthodont* in Abstract - (Word variations have been searched) | **430** |
| **Cochrane Database of Systematic Reviews** | (chlor* OR mouthrinse OR mouthwash OR mouth-rinse OR "mouth rinse" OR mouth-wash OR "mouth wash") AND orthodont* in Record Title OR (chlor* OR mouthrinse OR mouthwash OR mouth-rinse OR "mouth rinse" OR mouth-wash OR "mouth wash") AND orthodont* in Abstract - (Word variations have been searched) | **1** |
| **Scopus** | TITLE-ABS ((chlor* OR mouthrinse OR mouthwash OR mouth-rinse OR "mouth rinse" OR mouth-wash OR "mouth wash") AND orthodont*) | **671** |
| **Web of Science™ Core Collection** | (chlor* OR mouthrinse OR mouthwash OR mouth-rinse OR "mouth rinse" OR mouth-wash OR "mouth wash") AND orthodont*(title) or (chlor* OR mouthrinse OR mouthwash OR mouth-rinse OR "mouth rinse" OR mouth-wash OR "mouth wash") AND orthodont*(abstract)  All databases. Timespan: All years. Search language=Auto | **722** |
| **EMBASE** | (chlor*:ab,ti OR mouthrinse:ab,ti OR mouthwash:ab,ti OR 'mouth rinse':ab,ti OR 'mouth wash':ab,ti) AND orthodont*:ab,ti | **458** |
| **ProQuest Dissertations and Theses Global** | title((chlor* OR mouthrinse OR mouthwash OR mouth-rinse OR "mouth rinse" OR mouth-wash OR "mouth wash") AND orthodont*) OR abstract((chlor* OR mouthrinse OR mouthwash OR mouth-rinse OR "mouth rinse" OR mouth-wash OR "mouth wash") AND orthodont*)Filters activated: Full text | **47** |

- **Supplementary Table 3.** Excluded records with reasons.

| **Excluded records** | **Reason** |
| --- | --- |
| Evaluation the effect of two mouthwashes on surface characterization of orthodontic wire and bracket and friction between bracket and wire [https://www.cochranelibrary.com/central/doi/10.1002/central/CN-01862708/full] | Protocol of a later published study |
| Castelló CA, Zamora-Martínez N, Paredes-Gallardo V, Tarazona-Álvarez B. Effect of mouthwashes on the force decay of polymeric ligature chains used for dental purposes: a systematic review and meta-analysis. BMC Oral Health. 2023 Aug 4;23(1):538. | Systematic review of in vitro studies |
| Chitra P, Prashantha GS, Rao A. Long-term evaluation of metal ion release in orthodontic patients using fluoridated oral hygiene agents: An in vivo study. J World Fed Orthod. 2019;8(3):107-111. | Non- Randomized Clinical Trial |
| Chitra P, Prashantha GS, Rao A. Effect of fluoride agents on surface characteristics of NiTi wires. An ex vivo investigation. J Oral Biol Craniofac Res. 2020 Oct-Dec;10(4):435-440. doi: 10.1016/j.jobcr.2020.07.006. | Non- Randomized Clinical Trial |
| Rajendran A, Sundareswaran S, Peediyekkal LV, Santhakumar P, Sathyanadhan S. Effect of oral environment and prescribed fluoride mouthwashes on different types of TMA wires - An in-vivo study. J Orthod Sci. 2019 May 23;8:8. | Non- Randomized Clinical Trial |
